# Prospective evaluation of indocyanine green fluorescence for anastomotic site selection in laparoscopic colorectal cancer surgery

**DOI:** 10.1016/j.clinsp.2026.101226

**Published:** 2026-10-27

**Authors:** Antonio Rocco Imperiale, Lucas Faraco Sobrado, Carlos Frederico Sparapan Marques, Caio Sérgio Rizkallah Nahas, Guilherme Cutait de Castro Cotti, Rossana Verônica Mendoza Lopez, Ulysses Ribeiro-Junior

**Affiliations:** Instituto do Câncer do Estado de São Paulo, Hospital das Clínicas, Faculdade de Medicina, Universidade de São Paulo (HCFMUSP), São Paulo, SP, Brazil

**Keywords:** Indocyanine green, Fluorescence imaging, Colorectal surgery, Anastomotic leakage, Perfusion assessment

## Abstract

**Background:**

Indocyanine Green (ICG) fluorescence imaging has emerged as a valuable tool for intraoperative assessment of bowel perfusion in colorectal surgery, with the potential to reduce anastomotic leakage. However, standardized protocols for its use remain undefined. This study aimed to evaluate the feasibility and clinical impact of ICG fluorescence imaging in laparoscopic colorectal resections.

**Methods:**

A prospective cohort of 60 patients undergoing laparoscopic sigmoidectomy or low anterior resection for colorectal cancer was analyzed. ICG was administered after high vascular ligation to guide anastomotic site selection. Perfusion was assessed using ICG fluorescence and the marginal arcade test. Primary outcomes were changes in anastomotic site and leakage rates; secondary outcomes included fluorescence time and its association with clinical variables.

**Results:**

The mean time to fluorescence appearance was 24.5 seconds (range 8–45), with no significant differences according to sex (p = 0.20), age (p = 0.24), or neoadjuvant therapy (p = 0.38). ICG imaging led to modification of the planned anastomotic site in 6.7% of cases (n = 4), none of which developed leakage. In two cases, discordance between ICG fluorescence and marginal arcade assessment was observed. Overall, anastomotic leakage occurred in 6.7% (n = 4), exclusively in patients who had received neoadjuvant radiotherapy (p = 0.04). No association was found between leakage and anastomotic height, fluorescence time, or marginal arcade findings. No adverse reactions to ICG were reported.

**Conclusion:**

ICG fluorescence imaging is a safe and feasible adjunct for intraoperative perfusion assessment that supports anastomotic site selection and may contribute to reducing anastomotic leakage. Further studies incorporating quantitative fluorescence metrics are warranted.

## Highlights

• ICG fluorescence safely assesses colonic perfusion in colorectal surgery.
• Fluorescence timing was not affected by patient-related factors.
• ICG changed anastomotic site in 6.7% of cases.
• No leaks occurred in cases with ICG-guided site modification.
• Anastomotic leakage was associated with prior radiotherapy.

## Introduction

In recent decades, advancements in surgical techniques, perioperative care, and multimodal oncologic management have significantly improved outcomes for patients with colorectal cancer. The adoption of minimally invasive approaches, including laparoscopic and robotic surgery, has further enhanced precision, safety, and recovery in colorectal resections.

Despite these progressions, anastomotic leakage remains one of the most serious postoperative complications, with rates varying from 3% to 20%, depending on the anastomotic location and patient-related factors^[^^1^^,^^2^ Leakage is associated with increased morbidity, mortality, prolonged hospitalization, and higher rates of local recurrence, placing a substantial burden on healthcare systems^[^^3^^,^^4^ Ensuring adequate perfusion at the anastomotic site is critical for healing; however, conventional intraoperative assessment relies on subjective parameters such as bowel appearance, marginal arcade pulsatility, and mesenteric vessel palpation, which are poorly standardized and lack reproducibility.

The introduction of near-infrared fluorescence angiography with Indocyanine Green (ICG) offers a real-time, objective method for assessing bowel perfusion. This technology enables surgeons to visualize and quantify microvascular blood flow intraoperatively, assisting in the selection of an optimal anastomotic site. This study aimed to evaluate the role of ICG fluorescence in intraoperative decision-making for anastomotic site selection in patients undergoing colorectal resection, as well as to assess fluorescence dynamics across different patient subgroups.

## Material and methods

This prospective study was conducted at the Cancer Institute of the University of São Paulo Medical School (ICESP-HCFMUSP) between November 2017 and January 2024. The study was approved by the institutional ethics committee (approval n° 4.385.502), and all participants provided written informed consent. The study was conducted and reported in accordance with the Strengthening the Reporting of Observational Studies in Epidemiology (STROBE) guidelines.

Patients aged 18-years or older who underwent laparoscopic sigmoid resection or low anterior resection for colorectal cancer were included, provided that the planned anastomotic site was located between 3 and 15 cm from the anal verge. Exclusion criteria included a known allergy to ICG or iodine contrast, pregnancy or lactation, and age over 85-years.

All surgeries were performed laparoscopically following standardized oncologic principles, including high ligation of the inferior mesenteric artery and vein and mobilization of the splenic flexure. After complete dissection, vascular ligation, splenic flexure mobilization, and rectal transection, the potential anastomotic site in the proximal colon was identified and marked. It was initially selected based on conventional criteria, such as adequate bowel mobility for a tension-free anastomosis and visible signs of sufficient vascularization. A 25 mg vial of ICG was diluted in 10 mL of sterile water, and a fixed 10 mg (4 mL) intravenous bolus was administered to all patients, regardless of body mass index, followed by a 10 mL saline flush. Real-time fluorescence imaging was performed using the PINPOINT® laparoscopic imaging system (Fig. 1). Perfusion was assessed visually based on the dynamic inflow of ICG fluorescence. Time to fluorescence appearance was defined as the interval between intravenous ICG injection and the first visible fluorescence of the colonic wall at the planned transection site. Qualitative perfusion assessment was performed by the operating surgeon based on the extent and homogeneity of fluorescence. If perfusion at the selected site was deemed inadequate, a more proximal site with better perfusion, as indicated by ICG fluorescence, was chosen and marked for the anastomosis.

**Fig. 1:**
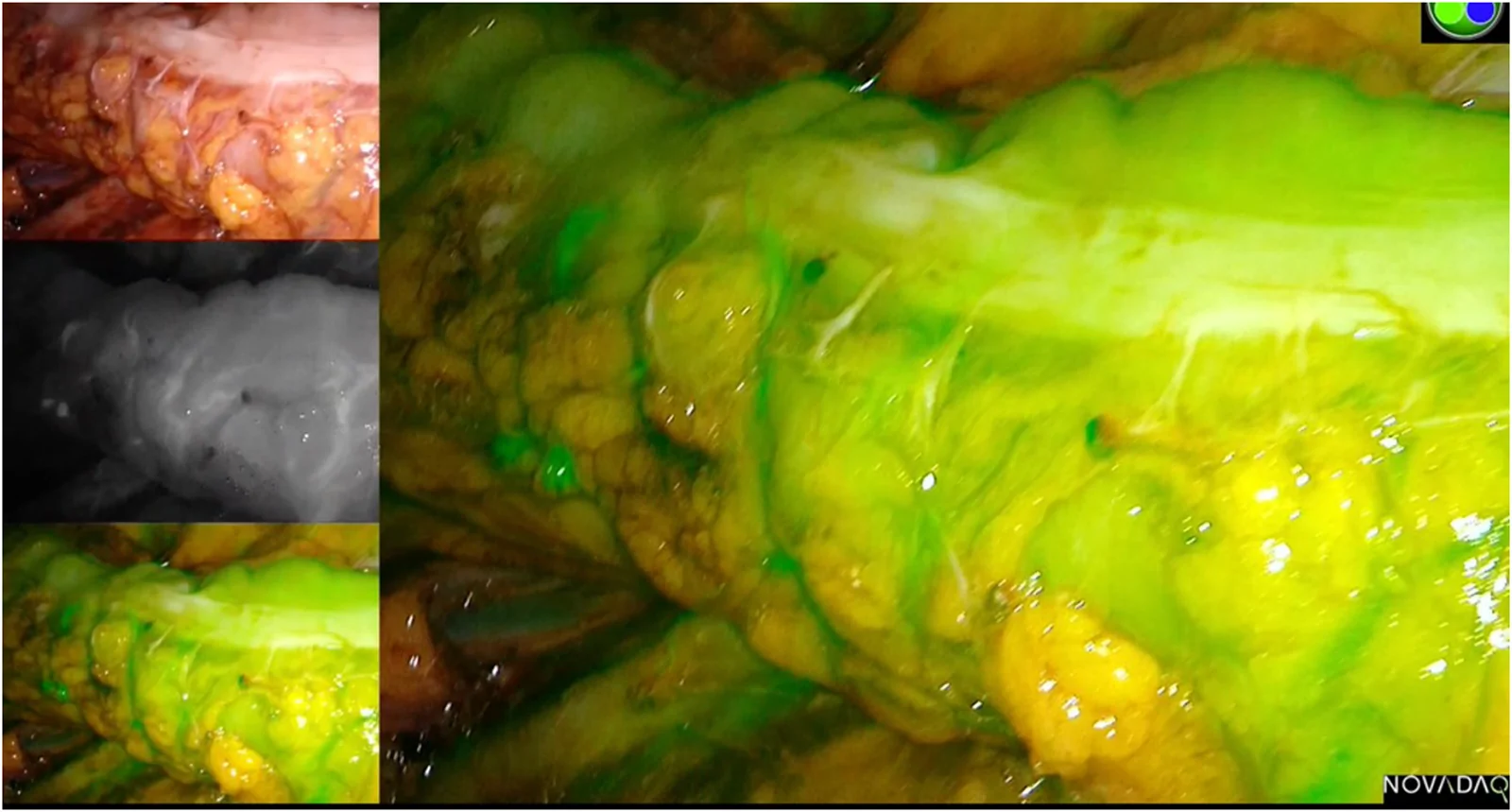
Intraoperative near-infrared fluorescence imaging using Indocyanine Green (ICG) during laparoscopic colorectal resection. Following intravenous administration of a 10 mg ICG bolus, real-time visualization demonstrates adequate colonic perfusion at the planned transection site using the PINPOINT® imaging system.

The specimen was exteriorized through a Pfannenstiel incision, and the marginal arcade was assessed at the marked site for bleeding, classified as good (pulsatile or active bleeding), regular (clearly visible but non-pulsatile bleeding), or poor (minimal bleeding). The marginal arcade test was performed only at the final anastomotic site after any adjustments based on ICG fluorescence, and was not systematically assessed at the initially selected (pre-ICG) site. Anastomosis was performed using a circular stapler, and its integrity was evaluated with an air leak test. At the conclusion of the procedure, a second evaluation of the anastomosis was performed via proctoscopy for all patients. An additional 10 mg dose of ICG was injected, and fluorescence appearance in the mucosa via the transrectal route was used to confirm the vascularization of both the anastomosis and the rectum (Fig. 2). A diverting ileostomy was routinely performed following TME according to the institutional protocol. This decision was independent of ICG findings. Low anastomoses were defined as those following TME, whereas high anastomoses were defined as those performed after partial mesorectal excision.

**Fig. 2:**
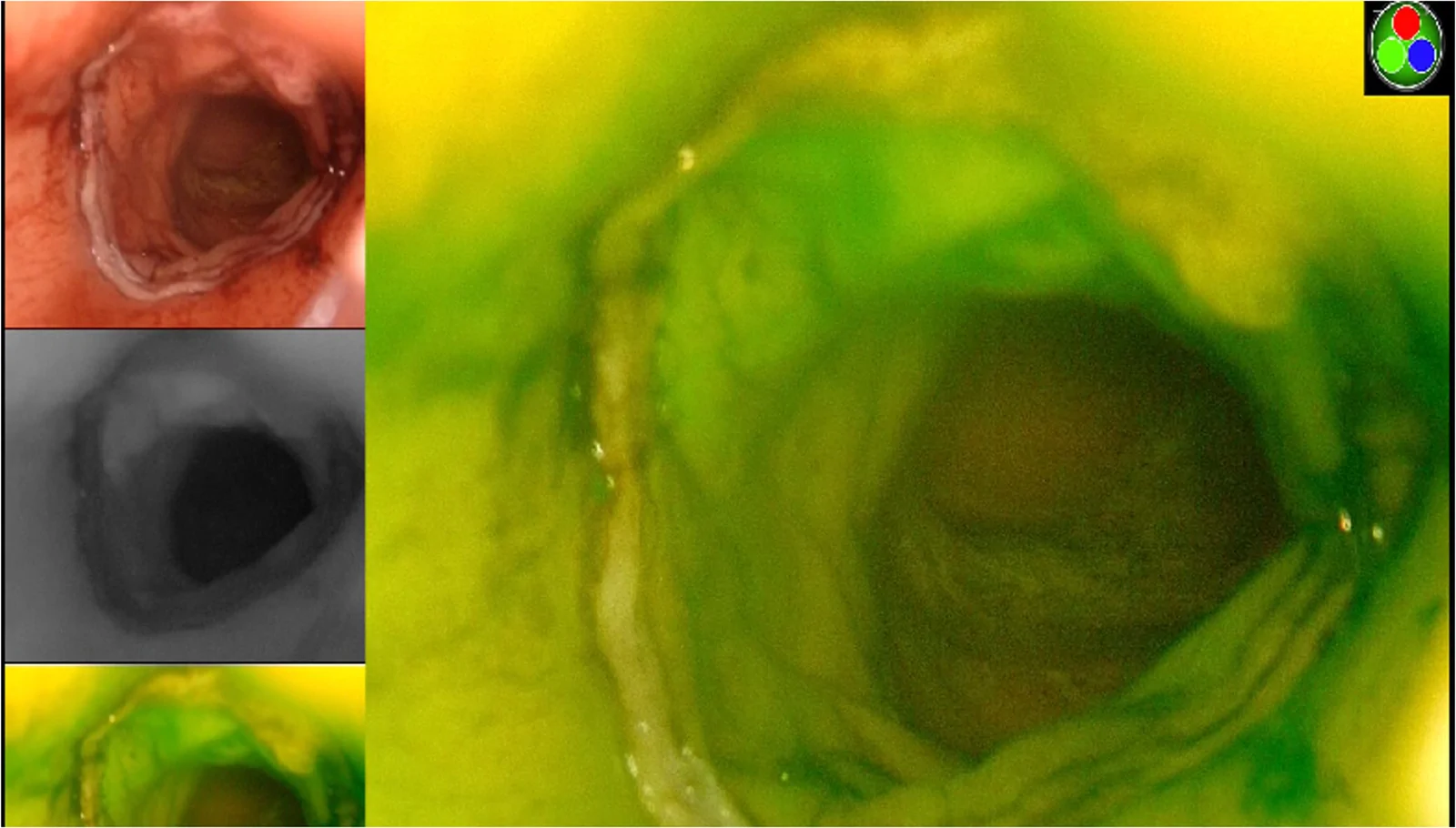
Transrectal fluorescence assessment of the colorectal anastomosis after intravenous administration of ICG. Proctoscopic evaluation demonstrates homogeneous fluorescence of the anastomosis and rectal stump, confirming adequate vascularization.

The primary outcomes of the study included the frequency of anastomotic site modifications based on ICG fluorescence findings and the incidence of anastomotic leakage. Secondary outcomes included the time to fluorescence appearance across subgroups defined by age, sex, and prior exposure to neoadjuvant therapy. For the age-based analysis, patients were categorized into three groups: younger than 50-years, 51 to 70 years, and older than 70-years. Data were analyzed using SPSS version 21 (IBM Corp.). Continuous variables were expressed as mean ± standard deviation, and statistical comparisons were made using Fisher’s exact test and Student’s *t*-test, with statistical significance set at p < 0.05.

## Results

A total of 60 patients underwent laparoscopic sigmoid or low anterior resection for colorectal cancer. The mean age was 59.8 ± 11.3 years, with 37 male patients (61.7%) and 23 female patients (38.3%). Neoadjuvant chemoradiotherapy was administered in 45% of cases (Table 1).

**Table 1:** Patient demographics and baseline characteristics (n = 60).

Variables	n (%)
Age, years, mean ± SD	59.8 ± 11.3
Female	23 (38.3)
Pre-operative Chemoradiation	27 (45)
Type of surgery	
Total mesorectal excision	20 (33.3)
Partial mesorectal excision	40 (66.7)
Operative time, minutes	272 (175 – 395)
Diverting Loop Ileostomy	20 (33.3)
Time to ICG fluorescence, seconds (range)	24.5 (8 – 45)
Changes in Planned Anastomotic Site	4 (6.7)
Marginal Artery Blood Flow	
Good	47 (78.3)
Regular	11 (18.3)
Poor	2 (3.3)
Leak Rates	4 (6.7)

Data are presented as n (%) unless otherwise indicated.

ICG, Indocyanine Green.

The mean time from ICG injection to fluorescence visualization was 24.5 seconds (range: 8–45 seconds). There were no statistically significant differences in fluorescence appearance time according to sex (p = 0.20), age group (p = 0.24), or prior exposure to chemoradiation (p = 0.38) (Table 2). ICG fluorescence led to modification of the initially planned anastomotic site in 6.7% of cases (n = 4). In all four patients, the transection line was moved to a more proximal site where adequate perfusion was demonstrated by ICG fluorescence, and none of these patients subsequently developed anastomotic leakage. Perfusion assessment using the marginal arcade test at the final anastomotic site revealed good flow in 47 patients (78.3%), regular flow in 11 (18.3%), and poor flow in 2 (3.3%). The anastomotic site was determined according to ICG fluorescence findings and was not modified following the marginal arcade assessment.

**Table 2:** Time to indocyanine green fluorescence appearance by sex, age group and exposure to neoadjuvant therapy.

Variables	Time, seconds	p-value
Sex		0.20
Male	24.0 ± 12.2	
Female	24.9 ± 6.7	
Age groups, years		0.24
31‒50	20.8 ± 4.8	
51‒70	25.5 ± 10.1	
> 70	25.1 ± 8.2	
Neoadjuvant chemoradiation		0.38
Yes	23.3 ± 6.4	
No	25.6 ± 10.8	

Data are presented as mean ± SD.

Low anastomoses were performed in 20 patients (33.3%), all with diverting loop ileostomy, while high anastomoses were performed in 40 patients (66.7%). Anastomotic leakage occurred in 6.7% of cases (n = 4), including two patients with low and two with high anastomoses. All patients who experienced leakage had undergone neoadjuvant radiotherapy. Among these four patients, three had good blood flow at the marginal arcade, and one had regular flow; all demonstrated homogeneous fluorescence on ICG assessment. Prior exposure to neoadjuvant chemoradiotherapy was the only factor significantly associated with anastomotic leakage (p = 0.04). Other factors, including sex (p = 0.29), anastomotic height (p = 0.59), age group (p = 0.999), and marginal arcade flow (p = 0.99), were not statistically significant. Mean fluorescence appearance time was similar between patients without and with leakage (24.5 ± 9.4 vs. 25.0 ± 4.1 seconds, p = 0.612), as was operative time (270.7 ± 51.8 vs. 288.8 ± 38.2 minutes, p = 0.441).

Three patients with anastomotic leakage were successfully managed with conservative treatment, including bowel rest and antibiotic therapy, whereas one required surgical reintervention with drainage and creation of a diverting ileostomy.

No adverse reactions or complications related to ICG administration were observed.

## Discussion

ICG fluorescence imaging has gained increasing recognition as a valuable tool in colorectal surgery, particularly for assessing bowel perfusion and supporting intraoperative decision-making aimed at reducing anastomotic leakage. Our study confirms the feasibility and safety of incorporating this technique without significantly altering the surgical procedure.

The timing and dosing of ICG administration have not been universally standardized, and prior studies have demonstrated variability in approach, with doses ranging from 0.2 mL/kg to 25 mg bolus [5, 6, 7, 8] In our study, we used a fixed 10 mg dose for all patients, regardless of body weight. This dosage, employed in several studies [9,10] has proven effective for ensuring clear perfusion visualization without excess fluorescence, which could compromise assessment [11]

While some studies administer ICG post-bowel transection, others do so before mesocolon ligation to guide anastomotic site selection. Our approach of injecting ICG after vascular ligation and before colonic transection allowed for early, real-time perfusion assessment, facilitating adjustments as needed. This strategy minimized procedural delays and enabled a direct comparison with the marginal arcade test, which, although traditionally accepted, remains subjective. However, a potential limitation of this approach is that perfusion assessed prior to transection may be influenced by collateral flow from mesocolic vessels beyond the marginal arcade, potentially overestimating perfusion at the selected site. In our series, post-anastomotic transrectal ICG assessment demonstrated adequate vascularization in all patients, suggesting preserved perfusion at the final anastomotic site.

Only one study has directly compared the marginal arcade test with fluorescence, concluding that fluorescence can be omitted when the marginal arcade appears sufficiently pulsatile [12] However, this study included a small sample size of just 20 cases. In contrast, in our study, despite two patients showing poor blood flow on the marginal arcade test, fluorescence imaging demonstrated good perfusion in all cases, which ultimately guided the anastomotic site selection. These findings suggest that ICG fluorescence may provide complementary information regarding tissue microcirculation beyond conventional macroscopic assessment. Recent evidence supports the correlation between ICG fluorescence and tissue oxygenation, providing a more objective perfusion assessment, as demonstrated by multisensor studies [13]

Our study found that fluorescence appearance time did not vary significantly by age, sex [5,7,9,14] or exposure to neoadjuvant therapy, which, to our knowledge, has not been reported before. Hagiwara et al.​ [15] found a significant association between delayed fluorescence and anastomotic leaks. In their cohort of 217 patients, those with leaks showed delayed fluorescence contrast by an average of 4 seconds, with a threshold of 31 seconds correlating with increased leakage risk. In contrast, we found no significant correlation between ICG visualization time and the occurrence of anastomotic leakage. Similarly, other studies have reported that visual assessment of timing parameters ‒ such as time to visibility, time to maximum visibility, and time to wash-out ‒ does not predict anastomotic leakage, with no significant differences between leak and non-leak groups [16]

This discrepancy may be explained by the subjective nature of visual fluorescence assessment, which may not capture subtle perfusion differences. Quantitative analysis may help standardize ICG perfusion assessment and improve the reliability of timing-based evaluation. However, its routine intraoperative applicability remains limited, as most studies rely on post hoc analysis, with only a minority performing real-time assessment to guide intraoperative decision-making [17]

The selection of anastomotic site based on fluorescence findings was reliable, with changes made in 6.7% of cases, consistent with previous reports ranging from 0% to 27.1% [18, 19, 20] Importantly, none of the patients whose anastomotic site was adjusted developed leakage, supporting the role of ICG fluorescence in optimizing anastomotic viability. Despite these advantages, four patients (6.7%) developed anastomotic leakage, all of whom had undergone neoadjuvant radiotherapy. This highlights the well-documented impact of radiotherapy on anastomotic healing, regardless of intraoperative perfusion assessment. Studies, including Shogan et al., have outlined the detrimental effects of radiation on collagen synthesis, tissue remodeling, and microvascular integrity, all of which can impair healing [21] This emphasizes the need for a multifactorial risk assessment that extends beyond real-time perfusion imaging.

The routine use of ICG and its impact on colorectal dehiscence continues to be debated. The PILLAR III study, the first randomized multicenter trial focused solely on colorectal cancer patients, was initially designed for 800 participants but was prematurely terminated after enrolling only 347 patients due to slow recruitment. Published in 2021, the study found no significant difference in anastomotic leak rates between the two groups [8] In contrast, the EssentiAL Trial, involving 850 patients from 41 hospitals across Japan, published in 2023, reported a decrease in anastomotic leak incidence from 11.8% to 7.6%, representing a relative reduction of 4.2% [22] These mixed findings highlight the need for further research to better define the role of ICG fluorescence in colorectal surgery. Its greatest value may lie in optimizing intraoperative decision-making in selected patients, rather than uniformly reducing anastomotic leakage across all colorectal resections.

It is also possible that colorectal anastomotic leaks may relate more to rectal perfusion than colonic perfusion, which led us to perform proctoscopy at the end of surgery. However, in all patients, both the anastomotic site and the residual rectum were well-vascularized. Although alterations in rectal stump perfusion have been implicated in anastomotic leakage [23] no such changes were observed in our cohort.

Our study has several limitations, including its single-center design, relatively small sample size, absence of a control group, and slow patient accrual during the COVID-19 pandemic. The absence of a control group limits causal inference regarding the effect of ICG fluorescence on anastomotic leakage. Because all patients underwent fluorescence assessment, the observed rate of anastomotic site modification and clinical outcomes cannot be directly compared with conventional visual assessment alone. Randomized controlled studies are therefore required to determine the magnitude of the clinical benefit attributable to ICG-guided perfusion assessment. Nonetheless, our study contributes to the existing literature by reaffirming the safety of ICG fluorescence, highlighting its dynamics across different subgroups, and demonstrating its potential to improve intraoperative assessment of colonic perfusion and guide anastomotic site selection. Previous studies have also shown the cost-effectiveness of this technique, supporting its routine use in colorectal surgery [24] Future advancements, such as quantitative fluorescence analysis, and artificial intelligence may further enhance the accuracy of ICG imaging and refine its role in surgical decision-making [25,26]

## Conclusion

ICG fluorescence is a safe and feasible adjunct for intraoperative assessment of colonic perfusion. It supports intraoperative decision-making during anastomotic site selection and may contribute to reducing anastomotic leakage. Further studies incorporating quantitative fluorescence metrics are warranted.

**Abbreviations**
ICG: Indocyanine Green
SPSS: Statistical Package for the Social Sciences
ICESP: Instituto do Câncer do Estado de São Paulo
HCFMUSP: Hospital das Clínicas da Faculdade de Medicina da Universidade de São Paulo
TME: Total Mesorectal Excision
COVID-19: Coronavirus Disease 2019

## Informed consent

Written informed consent was obtained from all individual participants included in this study.

## Authors’ contributions

All authors significantly contributed to the design of the study, data analysis, drafting, and approval of the final version of the manuscript. All authors agree to be accountable for all aspects of this study.

## Funding

None.

## Declaration of competing interest

The authors declare no conflicts of interest.

## Data Availability

The data generated during this study are available from the corresponding author upon reasonable request.
